# Detection of Mildewed Nutmeg Internal Quality during Storage Using an Electronic Nose Combined with Chemical Profile Analysis

**DOI:** 10.3390/molecules28166051

**Published:** 2023-08-14

**Authors:** Yang Cui, Yuebao Yao, Ruiqi Yang, Yashun Wang, Jingni Liang, Shaoqin Ouyang, Shulin Yu, Huiqin Zou, Yonghong Yan

**Affiliations:** School of Chinese Materia Medica, Beijing University of Chinese Medicine, Beijing 102488, China

**Keywords:** nutmeg, mildew, quality evaluation, odor characteristics, electronic nose, chemical profile

## Abstract

Internal mildewed nutmeg is difficult to perceive without cutting the nutmeg open and examining it carefully, which poses a significant risk to public health. At present, macroscopic identification and chromatographic analysis are applied to determine whether nutmeg is moldy or not. However, the former relies on a human panel, with the disadvantages of subjectivity and empirical dependence, whilst the latter is generally time-consuming and requires organic solvents. Therefore, it is urgent to develop a rapid and feasible approach for evaluating the quality and predicting mildew in nutmeg. In this study, the quality and odor characteristics of five groups of nutmeg samples with different degrees of mildew were analyzed by using the responses of an electronic nose combined with chemical profiling. The main physicochemical indicators, such as the levels of α-pinene, β-pinene, elemicin, and dehydro-di-isoeugenol, were determined. The results revealed that the contents of α-pinene, β-pinene, and elemicin changed significantly with the extension of storage time. Through the use of an electronic nose and HS–GC–MS technology to assess the overall odor characteristics of nutmeg samples, it was found that the production of volatile organic compounds (VOCs) such as ammonia/organic amines, carbon monoxide, ethanol, and hydrogen sulfide, as well as changes in the terpene and phenylpropene components of the nutmeg itself, may be the material basis for the changes in odor. The accuracy of the qualitative classification model for the degree of mildew in nutmeg was higher than 90% according to the electronic nose data combined with different machine learning algorithms. Quantitative models were established for predicting the contents of the chemical components, and models based on a BP neural network (BPNN), the support vector machine (SVM), and the random forest algorithm (RF) all showed good performance in predicting the concentrations of these chemical components, except for dehydro-di-isoeugenol. The BPNN performed effectively in predicting the storage time of nutmeg on the basis of the E-nose’s responses, with an RMSE and R^2^ of 0.268 and 0.996 for the training set, and 0.317 and 0.993 for the testing set, respectively. The results demonstrated that the responses of the electronic nose (E-nose) had a high correlation with the internal quality of nutmeg. This work proposes a quick and non-destructive evaluation method for the quality of nutmeg, which has high accuracy in discriminating between different degrees of mold in nutmeg and is conducive to early detection and warning of moldy phenomena.

## 1. Introduction

Nutmeg is a spice that is derived from the seed of the Myristica fragrans tree, which is native to Indonesian islands. Nutmeg has a warm, sweet, and slightly nutty flavor that makes it a popular ingredient in both sweet and savory dishes [1]. It is also used in traditional medicine for its digestive, anti-inflammatory, and analgesic properties [2]. According to a report by Statista, the global nutmeg market was valued at around USD 109.7 million in 2020 and is expected to reach USD 127.5 million by 2027. Nutmeg is commonly referred to as “the spice of life” [3] and has been used in traditional medicine for centuries because it contains several compounds that have potential health benefits, such as myristic acid, which has anti-inflammatory properties; and terpenes, which have antioxidant and anti-inflammatory effects [4]. Nutmeg contains 5–15% volatile oils, which include terpenes, phenylpropanoids, fatty acids, and miscellaneous compounds [5]. The main compounds of its essential oils are α-pinene, β-pinene, myristicin, elemicin, safrole, eugenol, and isoeugenol [6,7]. Among these compounds, α-pinene and β-pinene have been shown to have antimicrobial properties [8,9]. However, myristicin and elemicin are responsible for the hallucinogenic and intoxicating effects [10,11]. Regarding the spice, its medical value and safety must be accurately evaluated for quality during storage, and there is an urgent need to develop these evaluation methods.

The quality of nutmeg can deteriorate due to a variety of reasons, such as improper storage and processing periods, and exposure to moisture and high temperatures [12]. Nutmeg is susceptible to fungal contamination, which can cause the growth of mold and the subsequent synthesis of mycotoxins [13]. More alarmingly, mildew can grow in the interior of nutmeg, and it may not be visible to customers until it spreads to the surface. Additionally, some species of mildew can produce a powdery or fuzzy coating on the surface, which can easily be mistaken for natural nutmeg dust. Traditionally, detecting the quality of nutmeg, including the mold and degree of deterioration, has typically relied on sensory evaluations and physicochemical analyses. However, the results of sensory evaluations are subjective, as they can be influenced by the physical and psychological state of the evaluators. To obtain more objective and precise results, modern instruments such as gas chromatography–mass spectrometry (GC–MS), high-performance liquid chromatography (HPLC), and immunochromatographic assays can address these issues [14,15,16]. Although these chemical analysis methods offer accurate and dependable outcomes, the instruments required for detection can be costly, and the detection and analytical procedures are time-consuming and may damage the sample, making them impractical for real-time analyses. In recent years, various non-destructive techniques such as near-infrared spectroscopy, spectroscopic imaging, and thermal imaging have been applied for the detection of microbial contamination in agricultural products [17,18]. However, at the initial molding stage, there is no visible characteristic on the surface, which makes it challenging to detect the quality of nutmeg in a timely and real-time manner. Therefore, there is an urgent need for a device that can efficiently detect, quantify, and evaluate changes in the quality of nutmeg.

An electronic nose (E-nose) is a device that provides a fast, simple, and non-destructive method of analysis [19], and various types of E-nose have been designed and studied to identify and classify the aromatic compounds associated with biological and chemical changes in food and agricultural products during processing and storage [20]. Studies have been published that used E-noses to detect maturity in fruit and vegetables [21,22], grain shelf life [23], the degree of freshness of meat and fish [24,25], the authenticity of food products [26], etc. In evaluations of the quality of traditional Chinese medicine (TCM), gas sensor technology (E-nose) has demonstrated its potential application. When used in combination with various chromatographic techniques (such as GC–MS, HPLC, etc.), E-noses can help establish a correlation between the external characteristics of TCM and the changes in its internal components, which can help determine the material basis that affects the quality of TCM [27,28]. Some researchers have made attempts to detect the quality of nutmeg, such as developing a rapid method for predicting the total concentration of aflatoxin (AF) in nutmeg extracts using fluorescence fingerprinting (FF) [29]. Van Ruth et al. [30] found distinct differences between high-quality nutmeg and low-grade nutmeg samples in both their volatile and non-volatile fingerprints. These attempts focused on the effect of mycotoxins on nutmeg quality and the discrimination of different grades of nutmeg. However, no known previous study has focused on the quantitative detection of the quality indexes of nutmeg by applying an E-nose, such as detecting changes in the content of the main antibacterial components (α-pinene and β-pinene) during the process of mold growing nutmeg, as well as changes in the composition of the hallucinogenic substances due to prolonged storage.

As an objective and rapid detection method, the E-nose has shown promise as a substitute for traditional methods of evaluating the quality of nutmeg. This study mainly focused on developing a rapid and sensitive detection method for the qualitative and quantitative assessment of the quality of nutmeg by combining E-nose technology with chemical profile analyses, which can serve as a reference for monitoring and providing an early warning of mildewed medicinal materials. The specific objectives of this research were: (1) to clarify the changes in the physicochemical indexes (α-pinene, β-pinene, elemicin, and dehydro-di-isoeugenol) during the storage of nutmeg; (2) to find the chemical basis of the changes in the odor of nutmeg during the growth of mold; and (3) to qualitatively evaluate nutmeg samples under different molding conditions, and quantitatively predict the physicochemical indexes and storage time of nutmeg by applying E-nose signals based on chemometric methods, including the BP neural network (BPNN), support vector machine (SVM), random forest algorithm (RF), and partial least-squares regression (PLSR).

## 2. Results and Discussion

### 2.1. Changes in the Physicochemical Indexes of Nutmeg during Storage 

**Figure 1:**
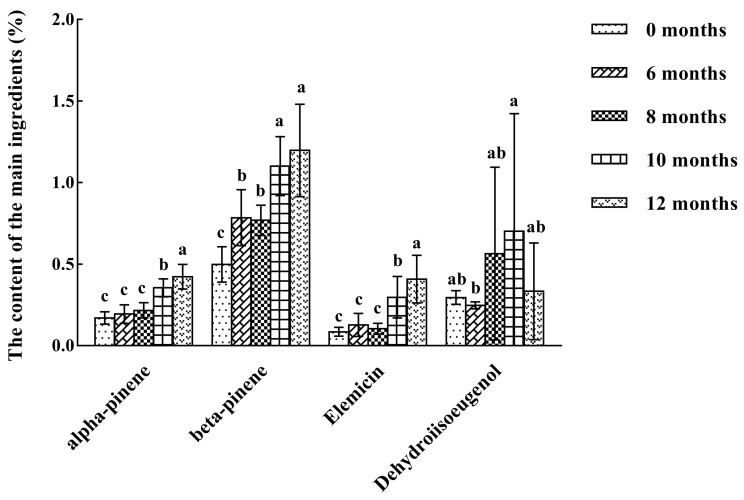
Changes in α-pinene, β-pinene, elemicin, and dehydro-di-isoeugenol during the storage of nutmeg. The letters a–c indicate significant differences in the means. Means with the same letters do not differ significantly.

Figure 1 shows the changes in α-pinene, β-pinene, elemicin, and dehydro-di-isoeugenol contents in nutmeg samples at different storage times. Nutmegs stored for 0 months contained 0.17% α-pinene, 0.49% β-pinene, 0.08% elemicin, and 0.29% dehydro-di-isoeugenol. The contents of α-pinene and β-pinene increased significantly by month 12 of storage (*p* < 0.05); the highest values were 0.42% and 1.19%, respectively. However, these results were different from those reported by Sanford and Heinz [31], showing that the source of the samples, storage environment, and experimental conditions will have an impact on the results. α-pinene and β-pinene are the main components of nutmeg’s volatile oil, which play an important role in the top aroma notes of nutmeg and have antibacterial and antioxidant effects [32,33], so an increase in their content may be related to nutmeg’s antibacterial effect. Yang et al. [34] discovered that the content of terpenes significantly decreased when nutmeg became moldy, but the changes in the content and mechanisms of the various components of terpenes still required further exploration. The content of elemicin generally increased with the extension of storage time, resulting in the highest value of 0.41% at month 12; a similar conclusion has been reported by Sanford and Heinz [31]. Elemicin has the effect of anti-liver lipid peroxidation, but also has hallucinogenic, anesthetic, and other toxic side effects. Therefore, when nutmeg is used as a condiment or cosmetic, its dosage should be strictly controlled.

Figure 1 illustrates that the contents of α-pinene, β-pinene, and elemicin had no significant difference (*p* < 0.05) at the initial stage of storage (from month 0 to month 8), indicating that the short storage period had no significant effect on these three indicators. The content of dehydro-di-isoeugenol conformed to the standard of the Chinese Pharmacopoeia (above 0.1%) in nutmegs with different degrees of mildew, but its content still had a downward trend with an increase in storage time. These results indicate that although nutmeg became mildewy at an early stage of storage, the content of the main indicator components did not change significantly. Therefore, it is necessary to combine other methods to evaluate the overall quality of nutmeg. The levels of α-pinene, β-pinene, elemicin, and dehydro-di-isoeugenol in all nutmeg samples measured by physicochemical tests are shown in Supplementary Table S1.

### 2.2. Odor Characteristics

**Figure 2:**
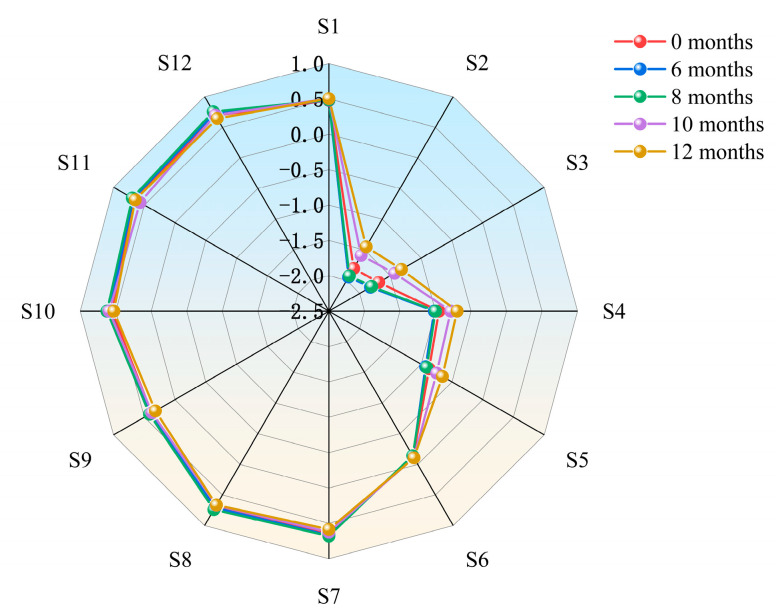
The radar map of the E-nose sensor responses for nutmeg during different storage periods.

The odor of nutmeg changed significantly during storage. The radar map (Figure 2) shows the changes in the response value of the E-nose during storage (0 months, 6 months, 8 months, 10 months, and 12 months). From Figure 2, it can be seen that the maximum response values of Sensors S2 and S3 were higher, followed by that of Sensor S4, and the maximum response value of Sensor S6 was lower. The most obvious changes were in Sensors S2, S3, S4, and S5, while the maximum response values of the remaining sensors almost overlapped in the radar map. The four significantly changed sensors are sensitive to ammonia/organic amines, carbon monoxide, ethanol, and hydrogen sulfide, respectively, indicating that these kinds of chemicals may be produced or changed in moldy nutmegs. Because the organic substances in nutmeg were decomposed by fungi during storage, the release of specific substances such as olefins, alcohols, aldehydes, and sulfide [35,36] could affect the flavor of food, and cause great damage to the quality of nutmeg.

**Figure 3:**
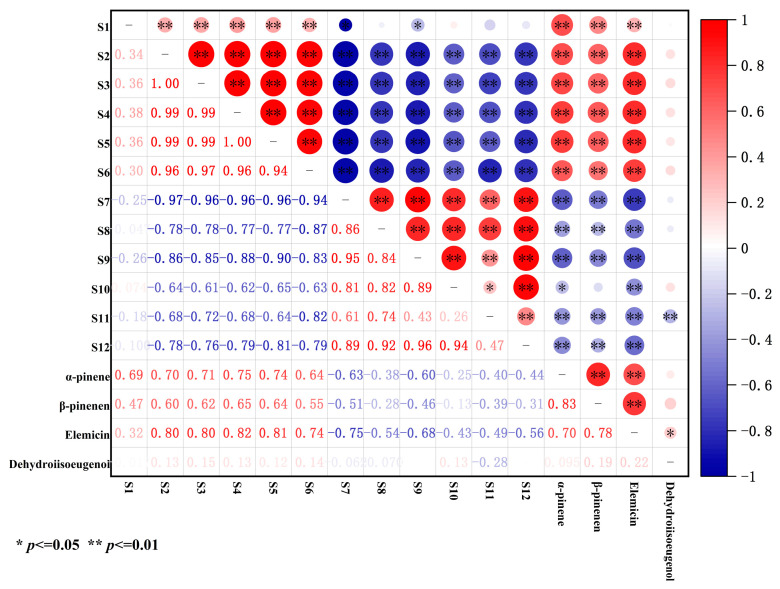
Pearson’s correlation analysis based on various indexes of nutmeg during storage. The color of a circle denotes the nature of the correlation, with 1 indicating a perfect positive correlation (dark red) and −1 indicating a perfect negative correlation (dark blue). Strong correlations and weak correlations are indicated by darker-colored circles and lighter-colored circles, respectively.

From Figure 3, it can be seen that there were significant linear negative correlations of α-pinene, β-pinene, and elemicin with S7 (organic solvents), S8 (hydrocarbons), S9 (methane), S10 (fluorine), S11 (aromatic compounds), and S12 (ethanol, ammonia, and organic amine). This indicates that the response values of the odor substances represented by these sensors increased with a decrease in α-pinene, β-pinene, and elemicin during the storage period of nutmeg. There is a highly linear positive correlation between α-pinene, β-pinene, and elemicin. However, dehydro-di-isoeugenol was almost unaffected by the odor substances and had no linear relationship with the other indicators, except for S11 (*p* < 0.01) and elemicin (*p* < 0.05).

### 2.3. PCA and OPLS-DA Analysis of Odor Ingredients

**Figure 4:**
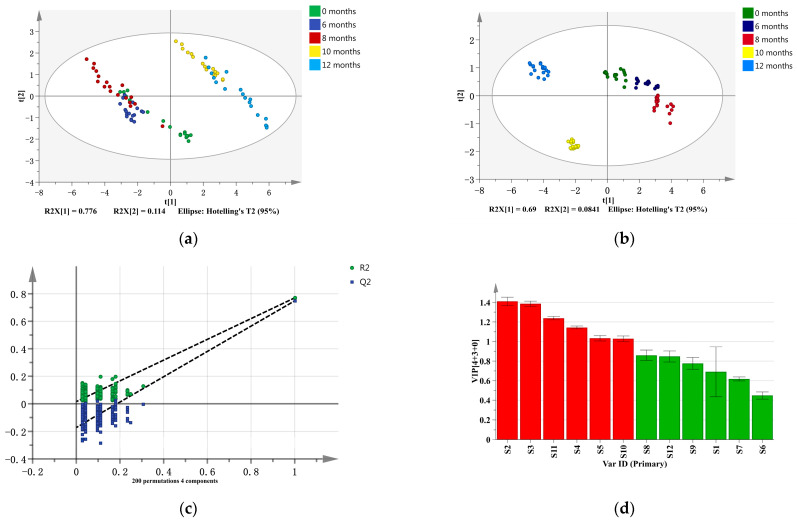
The results of the PCA plot: (**a**) the OPLS-DA plot; (**b**) the permutation test of OPLS-DA; (**c**) and the VIP value of the E-nose sensors in the analysis of nutmeg from different storage periods, i.e., 0 months, 6 months, 8 months, 10 months, and 12 months. Note: The red-highlighted part in (**d**) indicates sensors with VIP > 1.

In order to determine whether the data derived from the E-nose analysis were able to distinguish nutmeg samples with different storage times, PCA and OPLS-DA were used in this study. As shown in Figure 4a, PC1 and PC2 explained 77.6% and 11.4% of the total variance, respectively; both of the total variances were above 85%. However, there were overlapping areas among the nutmeg samples for 0 months, 6 months, and 8 months, as well as the nutmeg samples of 10 months and 12 months, indicating that the smells of these samples were similar. It is evident that the classification results were consistent with the changes in the physicochemical indexes of nutmeg. At the initial stage of storage, the changes in nutmeg odor and internal components are not obvious. However, with an extension of the storage time, the odor changed significantly, and the nutmeg samples stored for 0 months and 12 months could be clearly differentiated by the E-nose signals (Figure 4a).

Despite the clear separation observed in the PCA analyses, overlaps still existed. OPLS-DA comprises supervised statistical modeling tools that provide insights into the separations of nutmeg samples based on E-nose signals. The OPLS-DA model was supported by an R^2^X of 0.999, an R^2^Y of 0.815, and a Q^2^ of 0.799; R^2^ and Q^2^ exceeded 0.5, indicating that the model’s fitting results were acceptable. After 200 permutation tests, shown in Figure 4c, the intersection point of the Q^2^ regression line and vertical axis was less than zero, indicating that there was no overfitting in the model, and the model’s validation was effective. The samples of nutmeg from five storage periods could be clearly differentiated by the E-nose’s signals, as presented in Figure 4b. As depicted in Figure 4d, the variable importance in projection (VIP) values of Sensors S2, S3, S11, S4, S5, and S10 were all higher than 1. Regarding the results of analyzing the odor characteristics of nutmeg, Sensors S2, S3, S4, and S5 may be the key differential sensors for the nutmeg samples from five storage periods.

### 2.4. HS–GC–MS Analysis of the Volatile Organic Compounds of Nutmeg Samples 

**Figure 5:**
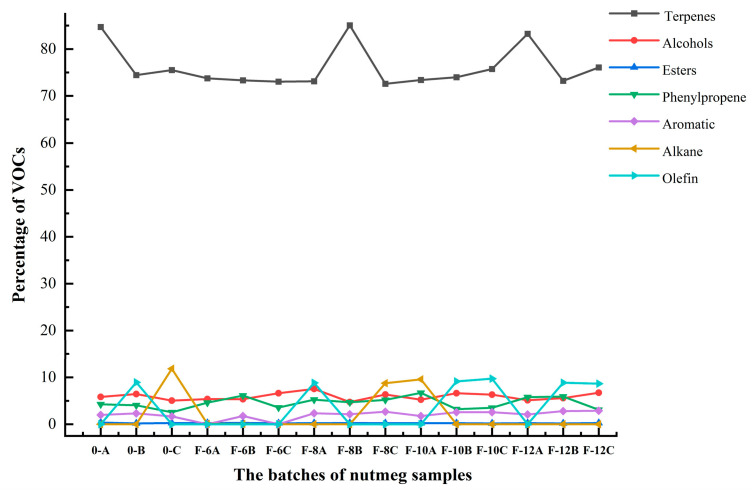
Trend chart of the proportion of various components of the volatile organic components (VOCs) of nutmeg. Note: A, B, and C represent three different batches of nutmeg.

Nutmeg possesses a distinct and strong aroma, which is attributed to the interaction of multiple chemical components. When nutmeg becomes moldy, its aroma undergoes changes, indicating alterations in its internal chemical components. HS–GC–MS was used to analyze the VOCs in nutmeg, with the aim of identifying the specific chemical constituents that caused the changes in odor. A detailed composition of the VOCs is shown in Supplementary Table S2. In total, 69 compounds were identified in all samples, mainly terpenes, phenylpropene, and alcohols. As the storage time extended, there was a downward trend in the terpene components of nutmeg, while the phenylpropene compounds and alcohols showed an upward trend (Figure 5). Monoterpenes contribute to the sweet and pleasant note of nutmeg. According to Supplementary Table S2, the relative abundance of sabinene was the highest during the initial month of storage (month 0), but its content decreased as the storage time extended. However, the relative content of myristicin and elemicin, which have woody and phenolic odors, increased with the prolongation of the storage time. The results indicated that the changes in the content of terpenes and phenylpropenes in nutmeg may be responsible for changes in the odor of nutmeg during the growth of mold.

### 2.5. Qualitative Classification of the Degree of Moldiness in Nutmeg Based on Different Machine Learning Algorithms

In order to find the most effective method for accurately evaluating nutmeg with different degrees of mildew, eight classifiers that were useable on Weka software 3.8.5 were implemented and compared in our research: the logistic classifier, ibk, Kstar, Multilayer Perception, random forest, J48, Bayes Net, and naïve Bayes. We used 10-fold cross-validation to evaluate the performance of all eight classification models. The odor response values of 81 nutmeg samples were used as training data, and the odor response values of 9 nutmeg samples were used for testing in each run. We recorded the accuracy, precision, recall, and F-measure scores of each classification model. The accuracy of classification was calculated as the number of correctly classified instances divided by the total number of instances. The F-measure is the harmonic average of the precision and recall [37,38,39]. Table 1 presents the rankings of these scores of the classifiers. The logistic classifier achieved the best values of accuracy (99.30%), precision (0.989), and recall (0.989), and the best F-measure (0.989). Therefore, the logistic classifier was selected as the machine learning algorithm with the highest performance. The Lazy.ibk and Lazy.Kstar classifiers had the same classification performance, with an accuracy of 95.80% and an F-measure of 0.934, second only to the logistic classifier. We found that E-nose data combined with a machine learning algorithm could accurately classify different degrees of mildew in nutmeg, which is conducive to giving warnings about the quality of nutmeg.

### 2.6. Quantification of the Predicted Physicochemical Indexes of Nutmeg

Different algorithms were applied to establish regression models to predict the content of chemical components, including α-pinene, β-pinene, elemicin, and dehydro-di-isoeugenol, based on the E-nose signals. Seventy percent of the data were used for training and 30% were used for testing, so the data from 90 nutmeg samples were divided into the training set (63 samples) and the testing set (27 samples). The squared correlation coefficient (R^2^) and the root mean square error (RMSE) were used as internal indicators of the stability and the predictive ability of the models. In general, a good prediction model always has high R^2^ values and low RMSE values.

The results are shown in Table 2, which shows the unsatisfactory performance of the four different algorithms for predicting the dehydro-di-isoeugenol content (R^2^ ≤ 0.6126, RMSE ≥ 0.2623), with a low R^2^ and a high RMSE. The results were consistent with the results of the correlation analysis above, which showed that dehydro-di-isoeugenol was almost unaffected by the odor compounds, so using E-nose data alone is not suitable for predicting the content of non-volatile constituents. We found that elemicin was predicted well, except by the PLSR algorithm. For independent E-nose signals, the R^2^ and RMSE values for elemicin based on BPNN were R^2^ = 0.9450 in the training set and R^2^ = 0.9579 in the testing set; those based on SVM were R^2^ = 0.9103 in the training set and R^2^ = 0.8831 in the testing set; and those based on RF were R^2^ = 0.9287 in the training set and R^2^ = 0.8772 in the testing set. The results showed a good correlation between the E-nose data and the predicted value of elemicin (R^2^ ≥ 0.9103 in the training set, R^2^ ≥ 0.8772 in the testing set). The prediction model based on BPNN had the best performance compared with the models based on SVM and RF. However, the performance of the PLSR model was unsatisfying for predicting elemicin (R^2^ was only about 0.7 in the training and testing sets). The results indicated that BPNN models based on independent E-nose signals could provide satisfactory performance in forecasting the content of elemicin.

Similar to the prediction of elemicin, the results obtained for the prediction of pinene components based on the PLSR regression models (R^2^ ≥ 0.6024 in the training set, R^2^ ≥ 0.6218 in the testing set) were worse than those of the other three algorithms. The R^2^ of the SVM and RF regression models for predicting α-pinene and β-pinene were greatly enhanced, and the RMSE values were decreased (SVM training set: R^2^ ≥ 0.8653, RMSE ≤ 0.1114; SVM testing set: R^2^ ≥ 0.8016, RMSE ≤ 0.1338; RF training set: R^2^ ≥ 0.8640, RMSE ≤ 0.1175; RF testing set: R^2^ ≥ 0.8092, RMSE ≤ 0.1142). Therefore, the prediction models based on SVM and RF were more accurate than those based on PLSR according to the statistical parameters (Table 2).

Via the BPNN, SVM, RF, and PLSR regression models, the E-nose’s ability to predict α-pinene, β-pinene, elemicin, and dehydro-di-isoeugenol values in nutmeg with different degrees of mildew was verified in this research. Generally, BPNN had superior performance for making predictions compared to SVM, RF, and PLSR, and achieved the best predictions for the content of elemicin. The results indicated that the E-nose data had better correlations with elemicin than with α-pinene, β-pinene, and dehydro-di-isoeugenol. The performance of the predictive model had to be improved, especially for predicting dehydro-di-isoeugenol.

### 2.7. Prediction of the Storage Time of Nutmeg

**Figure 6:**
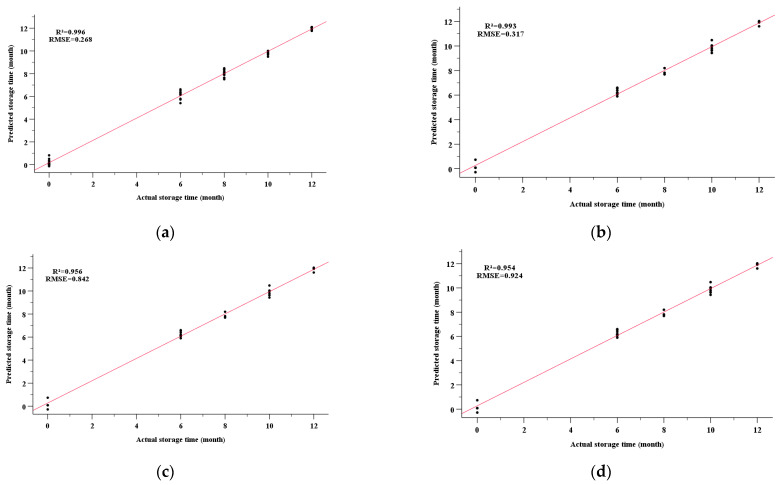
BPNN results for predicting the storage time of nutmeg on the basis of the E-nose signals ((**a**) training set; (**b**) testing set) and the physicochemical indexes ((**c**) training set; (**d**) testing set).

The storage times of the nutmeg samples were analyzed using BPNN, and the regression results are shown in Figure 6a–d. The BPNN model performed very effectively for predicting the storage time of nutmeg on the basis of the E-nose responses. The RMSE and R^2^ of the training set were 0.268 and 0.996, respectively; the results for the testing set were RMSE = 0.317 and R^2^ = 0.993. BPNN regression models were also set up to predict the storage time on the basis of the physicochemical indexes. As shown in Figure 6c,d, the RMSE and R^2^ of the training set were 0.842 and 0.956, respectively, and for the testing set, the results were RMSE = 0.924 and R^2^ = 0.954. The predicted results were inferior to when the E-nose responses were used.

Although nutmeg becomes moldy from the inside out, the volatile compounds can penetrate the shell, which can be quickly identified by an E-nose, providing the possibility for early monitoring and warning of mold in this type of medicinal material. BPNN regression models based on independent E-nose signals achieved the best predictions for nutmeg samples with different storage times.

## 3. Materials and Methods

### 3.1. Preparation of the Nutmeg Samples

In April 2019, three batches of nutmeg samples were purchased from Hebei Xinghua Traditional Chinese Medicine Co., Ltd. (Anguo City, China). To simulate the natural mildew of nutmeg, they were stored in Sanming City, Fujian Province (26°13′ N, 117°36′ E). The degree of mildew in nutmeg is related to its storage time. The test nutmegs were collected in December 2019 (this month was defined as month 0) and were sampled in June, August, October, and December 2020 (these months was defined as months 6, 8, 10, and 12, respectively). Hence, there were 15 nutmeg samples (3 batches and 5 sampling times) subsequently used for detection.

### 3.2. Setup of the Electronic Nose and Acquisition of Signals

An E-nose (α-Fox3000 E-nose, Alpha M.O.S., Toulouse, France) was used to obtain the odor characteristics of nutmeg. The E-nose contained three components: an automatic sampling system (HS-100 sampler), a sensor array, and a signal collecting system. The sensor array consisted of 12 metal oxide sensors; the names and response characteristics of each sensor are presented in Table 3. The E-nose was self-checked and the sensor array was preheated for 2–3 h before each sampling experiment. Processed pure air was used as the carrier gas to clean the sensor array, taking the signal response back to the baseline. We weighed 0.2 g of each sample and placed it in a 10 mL headspace vial. The E-nose program was set as follows: incubation time, 60 s; incubation temperature, 35 °C; oscillation speed, 250 r/min; injection volume, 300 μL; flow rate of the carrier gas, 150 mL/min; injection needle temperature, 45 °C; data acquisition time, 120 s; data acquisition interval, 1 s; cleaning time of the sensor array, 1080 s. Each nutmeg sample was measured in six replicates, and the maximum response values of the E-nose sensor were extracted and used for further analysis.

### 3.3. Analysis of the Chemical Profile of the Nutmeg Samples

#### 3.3.1. Main Chemicals and Reagents

HPLC-grade methanol was purchased from Thermo Fisher Technology (China) Co., Ltd. (Shanghai, China). The other reagents, including ethyl acetate, were of analytical grade and were purchased from Beijing Chemical Works (Beijing, China). Reference standards of dehydro-di-isoeugenol, α-pinene, and β-pinene were purchased from Shanghai Standard Technology Co., Ltd. (Shanghai, China); the reference standard of elemicin was purchased from Bioruler (Danbury, CN, USA). The purities of the reference standards were greater than 98.0%.

#### 3.3.2. GC–MS Analysis of the Contents of α-Pinene, β-Pinene, and Elemicin

An aliquot of 1.0 g of each nutmeg sample was extracted with 20 mL of ethyl acetate via ultrasonication at 40 kHz for 45 min. After cooling, the samples were weighed, and we used methanol to make up the lost weight. The processed samples were filtered and stored at 4 °C before analysis. All samples were analyzed in triplicate.

We accurately weighed a certain amount of the α-pinene, β-pinene, and elemicin reference standards and placed them in a 20 mL volumetric flask. We dissolved and diluted the standards with ethyl acetate to prepare a final mixed standard solution containing 2087.00 μg/mL of α-pinene, 1649.00 μg/mL of β-pinene, and 2038.00 μg/mL of elemicin. All the solutions were stored at 4 °C.

The GC–MS system included an Agilent 7890 B GC coupled to a 5977A mass spectrometer (Agilent Technologies, Santa Clara, CA, USA). Chromatography was performed on a HP-5MS 30 m × 0.25 mm × 0.25 μm column (Agilent Technologies, Santa Clara, CA, USA). The conditions of the GC–MS analysis were as previously described by Zhao et al. [5].

#### 3.3.3. HPLC Analysis for the Content of Dehydro-di-isoeugenol

The method of determining dehydro-di-isoeugenol was based on the method of the 2020 edition of the Chinese Pharmacopoeia. The detection of dehydro-di-isoeugenol was conducted via high-performance liquid chromatography (HPLC) (Shimadzu LC-20AT, Tokyo, Japan) on an apparatus equipped with a UV detector. The UV absorbance was monitored at 274 nm. The chromatographic separation was carried out on a ZORBAX Eclipse Plus-C18 column (4.6 mm × 250 mm, 5 μm). Methanol and water (75:25, *v*:*v*) were used as the solvent system for the HPLC analysis under the following conditions: flow rate, 1.0 mL/min; column temperature, 25 °C; injection volume, 10 μL.

#### 3.3.4. Analyses of the Volatile Organic Compounds

The volatile organic compounds (VOCs) of moldy nutmeg samples were determined using the HS–GC–MS system, which included an Agilent 7697A HS-7890B GC-5977MS (Agilent Technologies, Santa Clara, CA, USA). For this analysis, 0.5 g of each sample was weighed accurately and placed into a sample bottle with a 25 mL headspace, then incubated at 140 °C for 30 min. The VOCs were separated using a HP-5MS Ultra Inert column (30 m × 250 μm × 0.25 μm). The GC parameters were as follows: the initial oven temperature was 40 °C, which was held for 2 min, then ramped at 6 °C/min to 240 °C and held for 2 min. Helium carrier gas was used at a flow rate of 1 mL/min. The MS analysis was operated with the following parameters: the electrons’ impact mass spectra were recorded at an ionization energy of 70 eV by scanning the MS from *m*/*z* 40 to 450; the temperatures of the ionization source and the quadrupole were 230 °C and 150 °C, respectively. The VOCs were identified using the following methods: qualitative ion fragments acquired from the MS were analyzed with the mass spectral database NIST 14.L (NIST Library Search program), and hypothetical compounds with a match score of >80 were selected as the possible components of unknown compounds.

### 3.4. Statistical Analysis

Data are expressed as the mean ± standard deviation (SD). The differences between the groups with different storage times were analyzed via a one-way analysis of variance (ANOVA), with the significant difference set at *p* < 0.05 as per Duncan’s multiple tests. Statistical analysis was performed using SPSS version 20.0 (SPSS Inc., Chicago, IL, USA). Pearson’s rank correlation analysis between the physicochemical data and the E-nose data was performed using Origin 2021 software (Origin Lab, Northampton, MA, USA).

Unsupervised principal component analysis (PCA) and supervised orthogonal partial least-squares discriminant analysis (OPLS-DA) were performed using SIMCA software version 14.1 (Umetrics, Sweden) to visualize the differences among nutmeg samples from different storage periods. Nutmeg samples with different degrees of mildew were classified using WEKA 3.8.5 software http://www.cs.waikato.ac.nz/ml/weka/ (accessed on 24 December 2022), and eight classifiers (the logistic classifier, ibk, Kstar, Multilayer Perception, random forest, J48, Bayes Net, and naïve Bayes) were selected. Ten-fold cross-validation was used to evaluate the performance of all eight classification models. First, the data from all samples were randomly divided into 10 subsets. Each time, nine subsets of them were used as the training set and the remaining subset was the testing set. Finally, we calculated the parameters of accuracy, precision, recall, and the F-measure to evaluate the accuracy of the classification models.

The BP neural network (BPNN) is a relatively traditional backpropagation neural network algorithm that is widely used for prediction and classification. BPNN consists of an input layer, a hidden layer, and an output layer, all of which are connected. It is worth noting that the number of hidden layer nodes in the network is a key parameter that affects the accuracy of the network’s predictions [40]. Therefore, it is usually necessary to run BPNN multiple times to train the model to achieve the optimal number of hidden layer nodes. The support vector machine (SVM) is a supervised learning algorithm commonly used for classification and regression, which strives to fit the training data as closely as possible to a single model [41]. In the establishment of SVM models, the kernel functions most commonly used include Gaussian kernel functions, radial basis functions (RBF), and sigmoid kernel functions. In this study, the SVM model used RBF as the kernel function. Random forest (RF) is an ensemble learning algorithm that integrates multiple models to make voting decisions in order to solve the defects caused by a single model [42]. It contains the classifiers of multiple decision trees, the results of which are jointly determined by the output of multiple decision trees. Partial least-squares regression (PLSR) is the most common method used for developing multivariable calibration models [43]. Matlab R2022b (Mathworks, Natick, MA, USA) was used to analyze the data, including with the BPNN, SVM, RF, and PLSR models.

## 4. Conclusions

In this study, the changes in both the physicochemical indexes and odor characteristics of nutmeg during storage were analyzed, and their correlations were identified. A negative linear correlation was observed between the changes in the physicochemical indexes (α-pinene, β-pinene, and elemicin) and the characteristic odor substances, including hydrocarbons, methane, ethanol, ammonia, and organic amines, which indicated that there is a strong correlation between the quality and odor characteristics of nutmeg. The degree of moldiness in nutmeg increases as the storage time is prolonged, and the nutmeg samples from five storage periods could be clearly differentiated via OPLS-DA based on E-nose signals. In this experiment, the variable importance in projection (VIP) values of S2, S3, S11, S4, S5, and S10 were all higher than 1. Regarding the results of analyzing the odor characteristics of nutmeg using E-nose technology, it has been suggested that Sensors S2, S3, S4, and S5 may be the key sensors for differentiating between the five storage periods. The four most significantly changed sensors are sensitive to ammonia/organic amines, carbon monoxide, ethanol, and hydrogen sulfide, respectively. When using HS–GC–MS technology to detect the volatile organic compounds of moldy nutmeg, it was found that there was a downward trend in the terpene components of nutmeg, while the phenylpropene and alcohol compounds showed an upward trend. In summary, the production or alteration of these volatile organic compounds may be the material basis for the changes in odor during the process of nutmeg becoming moldy. In addition, the degree of moldiness in nutmeg can be qualitatively classified by different machine learning algorithms. The logistic classifier was selected as the machine learning algorithm with the highest performance, which achieved the best accuracy (99.30%), precision (0.989), recall (0.989), and F-measure (0.989).

For quantitative prediction, BPNN, SVM, RF, and PLSR were applied, based on the E-nose signals, to predict the chemical components (α-pinene, β-pinene, elemicin, and dehydro-di-isoeugenol) and storage periods. The performance of the prediction models using different algorithms based on electronic signals was compared. It was demonstrated that elemicin was predicted well, except by the PLSR algorithm. BPNN achieved the best predictions for the content of elemicin. Meanwhile, the prediction models for α-pinene and β-pinene based on SVM and RF were more accurate than those based on PLSR according to the statistical parameters. However, the performance of all four algorithms was unsatisfactory for predicting dehydro-di-isoeugenol, with a low R^2^ and a high RMSE. Using E-nose data alone is not suitable for predicting the content of involatile constituents. The BPNN performed very effectively for predicting the storage period on the basis of the E-nose responses and the physicochemical indexes.

This study showed that the application of E-nose technology combined with appropriate algorithms could be successfully used in the qualitative and quantitative detection of the quality of nutmeg, which can serve as a reference for monitoring and early warning of mildew in medicinal materials.

## Figures and Tables

**Table 1:** Rankings of the F-measure of classifiers.

Rank	Classifier	Accuracy (%)	Precision	Recall	F-Measure
1	Functions.Logistic	99.30%	0.989	0.989	0.989
2	Lazy.ibk	95.80%	0.936	0.933	0.934
3	Lazy.Kstar	95.80%	0.935	0.933	0.934
4	Functions.Multilayer Perception	95.15%	0.944	0.922	0.926
5	Trees.Random Forest	94.45%	0.919	0.911	0.913
6	Trees.J48	93.05%	0.89	0.889	0.888
7	Bayes.Bayes Net	91.70%	0.88	0.867	0.869
8	Bayes.Naïve Bayes	91.00%	0.854	0.856	0.854

**Table 2:** The results of evaluating parameters for prediction models based on BPNN, SVM, RF, and PLSR.

Algorithm	Physicochemical Index	Training Set		Testing Set	
		R^2^	RMSEC	R^2^	RMSEP
BPNN	α-pinene	0.8878	0.0371	0.8821	0.0509
	β-pinene	0.7969	0.1357	0.8080	0.1332
	Elemicin	0.9450	0.0352	0.9579	0.0339
	Dehydro-di-isoeugenol	0.5927	0.2623	0.5284	0.3333
SVM	α-pinene	0.9348	0.0290	0.8276	0.0447
	β-pinene	0.8653	0.1114	0.8016	0.1338
	Elemicin	0.9103	0.0413	0.8831	0.0617
	Dehydro-di-isoeugenol	0.6126	0.2788	0.3796	0.3107
RF	α-pinene	0.9140	0.0332	0.8399	0.0439
	β-pinene	0.8640	0.1175	0.8092	0.1142
	Elemicin	0.9287	0.0421	0.8772	0.0511
	Dehydro-di-isoeugenol	0.5630	0.3082	0.3981	0.2699
PLSR	α-pinene	0.8074	0.0515	0.7941	0.0449
	β-pinene	0.6024	0.1923	0.6218	0.1843
	Elemicin	0.7048	0.0857	0.6959	0.0815
	Dehydro-di-isoeugenol	0.1845	0.4094	0.1611	0.3552

**Table 3:** Detailed information of 12 metal oxide sensors (α-Fox3000 E-nose, Alpha M.O.S., Toulouse, France).

No.	Type of Sensor	Sensitive Substance
S1	LY2/LG	Oxidizing gas
S2	LY2/G	Ammonia, carbon monoxide
S3	LY2/AA	Ethanol
S4	LY2/GH	Ammonia/organic amine
S5	LY2/gCTL	Hydrogen sulfide
S6	LY2/gCT	Propane/butane
S7	T30/1	Organic solvents
S8	P10/1	Hydrocarbons
S9	P10/2	Methane
S10	P40/1	Fluorine
S11	T70/2	Aromatic compounds
S12	PA/2	Ethanol, ammonia/organic amine

## Data Availability

Data are contained within the article and Supplementary Materials.

## Supplementary Materials

The following supporting information can be downloaded at https://www.mdpi.com/article/10.3390/molecules28166051/s1, Supplementary Table S1: The four main physicochemical indexes of nutmeg were investigated (*n* = 3). Supplementary Table S2: The volatile compounds of nutmeg identified via HS–GC–MS (relative content, %).
