# Supplementary material for: Detection of Mildewed Nutmeg Internal Quality during Storage Using an Electronic Nose Combined with Chemical Profile Analysis
Source: Molecules. 2023 Aug 14;28(16):6051. doi: 10.3390/molecules28166051 (PMC10457796; doi:10.3390/molecules28166051)
Supplement: Supplementary file 1 [file molecules-28-06051-s001.zip › molecules-2505284-supplementary.pdf]

Supplementary Table S1.

The four main physicochemical indexes of nutmeg were investigated (n = 3)

**Storage time**	**Content (%, mean±SD)**			
	**α-pinene**	**β-pinene**	**Elemicin**	**Dehydrodiisoeugenol**
0 months	0.170±0.038	0.499±0.109	0.085±0.027	0.295±0.041
6 months	0.194±0.056	0.785±0.172	0.127±0.070	0.248±0.021
8 months	0.216±0.047	0.768±0.092	0.104±0.033	0.565±0.529
10 months	0.355±0.055	1.101±0.180	0.297±0.127	0.702±0.722
12 months	0.422±0.075	1.197±0.284	0.408±0.147	0.334±0.297

Supplementary Table S2.

The volatile compounds of nutmeg identified by HS-GC-MS (relative content, %)

No.	Category	Compound	0-A	0-B	0-C	6-A	6-B	6-C	8-A	8-B	8-C	10-A	10-B	10-C	12-A	12-B	12-C
1	Terpenes	Cyclene	0.03	0.03	0.04	0.04	0.03	0.03	0.03	0.04	0.04	0.03	0.03	0.04	0.04	0.03	0.03
2		alpha-thujene	6.19	8.06	7.71	7.40	7.08	8.02	7.40	7.89	8.08	6.29	7.42	7.87	7.80	8.36	8.55
3		Cyclofenchene	16.01	12.52	14.16	12.80	15.23	11.33	14.16	15.01	13.42	15.43	13.34	15.88	15.51	14.07	14.96
4		Camphene	0.53	0.52	0.59	0.53	0.58	0.52	0.58	0.59	0.61	0.55	0.53	0.63	0.57	0.58	0.55
5		Sabinene	29.42	29.57	28.26	31.14	26.29	27.83	21.26	28.83	25.06	28.95	27.46	25.71	28.81	22.82	24.67
6		(-)-beta-Pinene	6.52	4.01	4.51	3.28	5.80	3.37	6.24	4.97	4.46	5.58	4.66	5.41	5.12	5.42	5.58
7		β-myrcene	4.74	4.24	4.42	4.29	4.16	4.16	3.94	4.36	4.04	4.16	4.05	3.91	4.18	3.97	4.10
8		α-phellandrene	1.14	1.66	2.33	2.09	1.77	2.37	2.03	1.88	2.02	1.51	1.84	1.93	1.41	2.17	1.82
9		(+)-4-Carene	3.32	4.58	4.45	3.75	3.91	5.01	5.84	3.99	4.75	3.29	4.59	4.77	3.87	5.52	5.59
10		γ-Terpinene	4.53	5.90	5.79	4.88	5.18	6.29	7.51	4.95	6.06	4.48	5.97	6.05	4.99	6.71	6.77
11		Terpinolene	1.66	2.26	2.20	2.12	2.15	2.74	3.08	1.85	2.66	1.67	2.52	2.39	1.94	2.74	2.47
12		.alfa.-Copaene	0.43	0.44	0.46	0.39	0.42	0.42	0.27	0.44	0.41	0.46	0.35	0.46	0.28	0.25	0.39
13		3-Carene	0.06	0.03	0.00	0.05	0.03	0.04	0.04	0.08	0.05	0.05	0.04	0.03	0.04	0.04	0.04
14		D-Limonene	9.25	0.00	0.00	0.00	0.00	0.00	0.00	9.64	0.00	0.00	0.00	0.00	8.30	0.00	0.00
15		β-ocimene	0.07	0.00	0.00	0.00	0.00	0.00	0.00	0.00	0.00	0.00	0.00	0.00	0.00	0.00	0.00
16		(-)-Germacrene-D	0.06	0.04	0.04	0.00	0.00	0.00	0.03	0.04	0.04	0.05	0.00	0.04	0.03	0.00	0.03
17		Caryophyllene	0.45	0.09	0.00	0.24	0.11	0.11	0.18	0.00	0.21	0.34	0.28	0.14	0.00	0.12	0.06
18		Humulene	0.08	0.00	0.00	0.04	0.00	0.00	0.00	0.00	0.04	0.04	0.00	0.00	0.00	0.00	0.00
19		beta-copaene	0.09	0.00	0.00	0.00	0.00	0.04	0.00	0.04	0.00	0.00	0.03	0.00	0.00	0.00	0.00
20		.gamma.-Elemene	0.03	0.00	0.03	0.00	0.00	0.00	0.00	0.00	0.00	0.00	0.00	0.00	0.00	0.00	0.00
21		.beta.-Bisabolene	0.03	0.00	0.00	0.00	0.00	0.00	0.00	0.00	0.00	0.00	0.08	0.00	0.00	0.00	0.04
22		Copaene	0.00	0.26	0.24	0.26	0.29	0.26	0.00	0.28	0.30	0.31	0.32	0.28	0.00	0.00	0.26
23		cis-.alpha.-Bergamotene	0.00	0.04	0.05	0.04	0.07	0.06	0.08	0.03	0.05	0.05	0.07	0.06	0.04	0.05	0.07
24		beta-Cubebene	0.00	0.18	0.11	0.41	0.12	0.28	0.10	0.00	0.10	0.08	0.33	0.10	0.06	0.06	0.05
25		(+)-3-Carene	0.00	0.00	0.05	0.00	0.00	0.05	0.00	0.10	0.00	0.04	0.00	0.00	0.00	0.00	0.00
26		(+)-delta-Cadinene	0.00	0.00	0.07	0.00	0.00	0.00	0.00	0.00	0.00	0.00	0.00	0.00	0.00	0.00	0.00

27	trans-Sesquisabinene hydrate	0.00	0.00	0.00	0.00	0.00	0.00	0.30	0.00	0.00	0.00	0.00	0.00	0.00	0.24	0.00
28	cis-.beta.-Farnesene	0.00	0.00	0.00	0.00	0.00	0.05	0.00	0.00	0.00	0.04	0.00	0.00	0.00	0.00	0.03
29	(E)-.beta.-Famesene	0.00	0.00	0.00	0.00	0.06	0.00	0.00	0.04	0.00	0.00	0.00	0.00	0.00	0.00	0.00
30	beta-elemene	0.00	0.00	0.00	0.00	0.00	0.03	0.00	0.00	0.00	0.00	0.03	0.00	0.00	0.00	0.00
31	alpha-Bergamotene	0.06	0.00	0.00	0.00	0.00	0.00	0.00	0.00	0.00	0.00	0.00	0.00	0.00	0.00	0.00
32	bicyclogermacrene	0.00	0.00	0.00	0.00	0.00	0.00	0.00	0.00	0.00	0.00	0.00	0.00	0.00	0.00	0.00
33	(Z)-β-ocimene	0.00	0.00	0.00	0.00	0.00	0.00	0.00	0.00	0.00	0.00	0.00	0.00	0.00	0.00	0.00
34	(1S,4aR,8aS)-1-Isopropyl-7-methyl-4-methylene-1,2,3,4,4a,5,6,8a-octahydronaphthalene	0.00	0.00	0.00	0.00	0.03	0.00	0.00	0.00	0.00	0.00	0.00	0.00	0.00	0.00	0.00
35	5,8-Dimethylenebicyclo[2.2.2]oct-2-ene	0.00	0.00	0.00	0.00	0.00	0.00	0.00	0.00	0.17	0.00	0.00	0.00	0.00	0.00	0.00
36	Germacrene B	0.00	0.00	0.00	0.00	0.00	0.00	0.00	0.00	0.00	0.00	0.04	0.00	0.00	0.00	0.00
37	Cyclohexene, 4-ethenyl-4-methyl-3-(1-methylethenyl)-1-(1-methylethyl)-, (3R-trans)-	0.00	0.00	0.00	0.00	0.00	0.00	0.00	0.00	0.00	0.00	0.00	0.00	0.25	0.00	0.00
38	Bicyclo[7.2.0]undec-4-ene, 4,11,11-trimethyl-8-methylene-,[1R-(1R*,4Z,9S*)]- (Note: the asterisk (*) indicates that there is a chiral center at that position)	0.00	0.00	0.00	0.00	0.00	0.00	0.04	0.00	0.00	0.00	0.00	0.03	0.00	0.03	0.00

39	Alcohols	2-Cyclohexen-1-ol, 1-methyl-4-(1-methylethyl)-, cis-	0.15	0.21	0.17	0.17	0.15	0.23	0.22	0.16	0.22	0.15	0.22	0.18	0.16	0.19	0.21
40		3-Cyclohexen-1-ol, 4-methyl-1-(1-methylethyl)-, (R)-	3.24	3.98	3.21	3.06	3.37	4.26	5.07	2.80	4.21	3.06	4.19	3.87	3.11	4.06	4.21
41		.alpha.-Terpineol	0.47	0.44	0.38	0.41	0.44	0.51	0.64	0.39	0.53	0.39	0.50	0.44	0.38	0.46	0.44
42		4-Thujanol	1.46	1.65	1.21	1.52	1.27	1.49	0.00	1.28	1.25	1.48	1.55	0.00	1.34	0.00	0.00
43		2-Cyclohexen-1-ol, 3-methyl-6-(1-methylethyl)-, trans-	0.05	0.07	0.05	0.05	0.06	0.08	0.10	0.05	0.07	0.05	0.00	0.00	0.05	0.09	0.07
44		gamma-terpineol	0.14	0.06	0.00	0.11	0.06	0.04	0.00	0.05	0.06	0.12	0.09	0.03	0.08	0.00	0.00
45		2-Cyclohexen-1-ol, 1-methyl-4-(1-methylethyl)-, trans-	0.00	0.00	0.00	0.00	0.00	0.00	0.00	0.00	0.00	0.00	0.00	0.00	0.00	0.00	0.00
46		cis-Piperitol	0.00	0.00	0.00	0.00	0.00	0.00	0.00	0.00	0.00	0.00	0.08	0.06	0.00	0.00	0.00
47		trans-4-thujanol	0.00	0.00	0.00	0.00	0.00	0.00	0.70	0.00	0.00	0.00	0.00	0.88	0.00	0.78	0.88
48		4-Thujanol	0.00	0.00	0.00	0.00	0.00	0.00	0.81	0.00	0.00	0.00	0.00	0.84	0.00	0.00	0.92
49		1-Naphthalenol, 1,2,3,4,4a,7,8,8a-octahydro-1,6-dimethyl-4-(1-methylethyl)-, [1R-(1.alpha.,4.beta.,4a.beta.,8a.beta.)]-	0.34	0.00	0.00	0.00	0.00	0.00	0.00	0.00	0.00	0.00	0.00	0.00	0.00	0.00	0.00
50		Citronellol	0.00	0.03	0.00	0.00	0.00	0.00	0.00	0.00	0.00	0.00	0.00	0.00	0.00	0.00	0.00
51		(2E,4S,7E)-4-Isopropyl-1,7-dimethylcyclodeca-2,7-dienol	0.00	0.00	0.00	0.04	0.00	0.00	0.00	0.00	0.00	0.00	0.00	0.00	0.00	0.00	0.00
52	Esters	(-)-Bornyl acetate	0.13	0.09	0.12	0.14	0.13	0.12	0.11	0.12	0.11	0.13	0.12	0.10	0.11	0.10	0.10
53		Neryl acetate	0.21	0.08	0.09	0.10	0.14	0.07	0.09	0.12	0.09	0.10	0.11	0.06	0.11	0.06	0.11

54		Linalyl acetate	0.00	0.00	0.04	0.00	0.00	0.00	0.03	0.00	0.00	0.00	0.00	0.00	0.00	0.03	0.04
55		Cyclohexanol,1-methyl-4-(1-methylethylidene)-, acetate	0.00	0.00	0.00	0.00	0.00	0.00	0.00	0.00	0.04	0.00	0.00	0.00	0.00	0.00	0.00
56	Phenylpr opene	Safrole	0.83	0.22	0.57	0.45	1.30	0.81	1.20	0.59	0.69	0.44	0.23	0.76	0.77	1.59	0.44
57		Methyleugenol	0.14	1.78	0.57	2.00	1.55	0.47	0.99	0.96	1.28	3.43	0.76	0.93	1.66	1.27	0.18
58		Myristicin	2.99	0.71	0.20	1.19	2.24	1.18	1.30	2.43	1.62	1.66	1.13	0.74	2.32	1.62	1.49
59		Elemicin	0.15	0.90	1.16	0.50	0.77	1.01	1.62	0.60	1.11	0.70	0.96	0.69	0.55	1.35	0.93
60		Phenol, 2-methoxy-3-(2-propenyl)	0.09	0.00	0.00	0.03	0.07	0.05	0.03	0.07	0.04	0.05	0.00	0.00	0.00	0.00	0.03
61		2,6-Dimethoxy-4-allylphenol	0.04	0.00	0.00	0.00	0.00	0.00	0.00	0.03	0.00	0.00	0.00	0.00	0.03	0.00	0.00
62		(E)-methyl isoeugenol	0.00	0.44	0.00	0.42	0.18	0.00	0.06	0.00	0.40	0.42	0.06	0.35	0.34	0.06	0.00
63		Methyl isoeugenol	0.00	0.00	0.04	0.00	0.00	0.00	0.00	0.00	0.00	0.00	0.00	0.00	0.00	0.00	0.00
64		Isoelemicin	0.00	0.00	0.00	0.00	0.00	0.04	0.06	0.00	0.03	0.00	0.03	0.05	0.00	0.00	0.00
65		γ-Asarone	0.00	0.00	0.00	0.00	0.00	0.00	0.00	0.00	0.00	0.00	0.03	0.00	0.00	0.00	0.00
66		Eugenol	0.00	0.00	0.00	0.00	0.00	0.00	0.00	0.00	0.00	0.00	0.00	0.00	0.08	0.00	0.00
67	Aromatic	o-Cymene	1.97	2.31	1.67	0.00	1.74	0.00	2.34	2.14	2.66	1.76	2.56	2.56	2.08	2.80	2.88
68	Alkane	Cyclohexane,1-methylene-4-(1-methylethenyl)-	0.00	0.00	11.83	0.06	0.05	0.00	0.00	0.00	8.77	9.57	0.00	0.00	0.00	0.00	0.00
69	Olefin	Cyclohexene, 1-methyl-5-(1-methylethenyl)-, (R)-	0.00	8.94	0.00	0.00	0.00	0.00	8.83	0.00	0.00	0.00	9.14	9.71	0.00	8.86	8.65
